# Clinical Utility of the OmniGraf Biomarker Panel in the Care of Kidney Transplant Recipients (CLARITY): Protocol for a Prospective, Multisite Observational Study

**DOI:** 10.2196/41020

**Published:** 2022-12-14

**Authors:** James N Fleming, Timothy Cober, Janelle Hickey, Leslie Stach, Allison Kawano, Amanda Szczepanik, Alicia Watson, Yuka Imamura, Juston Weems, Patricia West-Thielke

**Affiliations:** 1 Transplant Genomics, Inc Framingham, MA United States

**Keywords:** kidney transplant, biomarker, adverse event, adverse drug event, renal function, eGFR, clinical trial, allograft, nephrology, patient outcome, renal, kidney, transplant, observational study, medication monitoring, quality of life, chronic condition, medication management

## Abstract

**Background:**

Death with a functioning allograft has become the leading category of graft loss in kidney transplant recipients at all time points. Previous analyses have demonstrated that causes of death in kidney transplant recipients are predominated by comorbidities strongly associated with immunosuppressant medications. Adverse drug events (ADEs) have been strongly associated with nonadherence, health care utilization, and graft loss; clinicians face a difficult decision on whether making immunosuppressant adjustments in the face of ADEs will improve symptomology or simply increase the risk of acute rejection. Clinicians also face a treatment quandary in 50% of kidney transplant recipients with stage 3 or worse chronic kidney disease at 1 year post transplantation, as progressive decline in renal function has been strongly associated with inferior allograft survival.

**Objective:**

The primary objective of the CLinical Utility of the omnigrAf biomarkeR Panel In The Care of kidneY Transplant Recipients (CLARITY) trial is to evaluate change in renal function over time in kidney transplant recipients who are undergoing OmniGraf monitoring in conjunction with monitoring of their medication-related symptom burden (MRSB). A secondary objective of this study is to identify the impact of OmniGraf use in conjunction with patient-reported MRSB as part of clinical care on patients’ self-efficacy and quality of life.

**Methods:**

CLARITY is a 3-year prospective, multisite, observational study of 2000 participants with a matched control, measuring the impact of real-time patients’ MRSB and the OmniGraf biomarker panel on change in renal function over time. Secondary outcome measures include the Patient-Reported Outcomes Measurement Information System (PROMIS) Self-Efficacy for Managing Chronic Conditions–Managing Medications and Treatment–Short Form 4a; the PROMIS-29 Profile (version 2.1); the PROMIS Depression Scale, hospitalizations—subcategorized for hospitalizations owing to infections; treated rejections, MRSB, and proportion of participants with overall graft survival at year 3 post transplantation; graft loss or death during the 3-year study follow-up period; and change in provider satisfaction.

**Results:**

The primary outcome measure of the study will be a comparison of the slope change in estimated glomerular filtration rate from baseline to the end of follow-up between study participants and a matched control group. Secondary outcome measures include changes over time in PROMIS Self-Efficacy for Managing Chronic Conditions–Managing Medications and Treatment–Short Form 4a, the PROMIS-29 Profile (version 2.1), and PROMIS Depression Scale in the study group, as well as a comparison of hospitalizations and causes, rejections, and graft and patient survival compared between participants and a matched cohort. The anticipated first enrollment in the study is October 2022 with data analysis and publication expected in October 2027.

**Conclusions:**

Through this report, we describe the study design, methods, and outcome measures that will be utilized in the ongoing CLARITY trial.

**Trial Registration:**

ClinicalTrials.gov NCT05482100; https://clinicaltrials.gov/ct2/show/NCT05482100

**International Registered Report Identifier (IRRID):**

PRR1-10.2196/41020

## Introduction

The survival benefits of kidney transplantation in the United States are well documented [1,2]. Improvements in immunosuppression, better antimicrobial agents, and other aspects of ancillary care have resulted in significant improvements in short-term outcomes; however, there has been little improvement in long-term graft loss [3,4]. While the most recent Organ Procurement and Transplantation Network and Scientific Registry of Transplant Recipients Annual Data Review [5] shows that death-censored allograft failure has been improving at all time points, death with a functioning allograft has remained stable and is now responsible for more than half of graft losses at each time point. A multicenter analysis of specific causes of kidney allograft loss demonstrated that causes of death were predominated by comorbidities with strong associations with immunosuppressive medications, including cardiovascular and infectious diseases and cancers [6]. Further, a more recent analysis [7] found that 65% of kidney transplant recipients seeking hospital readmission had adverse drug events (ADEs) that were considered contributory, and ADE-associated readmissions had a significantly higher hazard of graft loss and death than readmissions without an ADE. ADEs have also been identified as a predictive factor for medication adherence in a large multicenter study. Couzi et al [8] also found that physicians significantly underestimated the prevalence of adverse events when compared to patient self-reporting. While assessments of the impact of real-time knowledge of adverse events on mutability of readmissions and outcomes are lacking, a randomized controlled trial [9] of a mobile health intervention that included real-time ADE tracking demonstrated significant reductions in hospitalizations and grade 3 or higher ADEs. Even with knowledge of ADEs, clinicians may be reluctant to adjust immunosuppressive medications owing to concerns of rejection risk during the period of medication adjustment.

Clinicians also find themselves in a clinical quandary when faced with patients with poor graft function. According to the most recent Organ Procurement and Transplantation Network and Scientific Registry of Transplant Recipients Annual Data Review [5], 50% of kidney transplant recipients have stage 3 chronic kidney disease (CKD) or worse at 1 year post transplantation. A large international analysis by the Patient Outcomes in Renal Transplantation investigators demonstrated that patients with stage 3b CKD or lower at 1 year post transplantation were at a significantly higher risk of graft failure by 10 years post transplantation—a risk that increased with a decrease in the estimated glomerular filtration rate (eGFR) [10]. Clinicians are aware of this, but they must also consider the risk of acute rejection that increases whenever immunosuppression is altered.

The OmniGraf biomarker panel (Transplant Genomics, Inc) includes the TruGraf peripheral blood expression profile and the Viracor TRAC donor-derived cell-free DNA test, which have demonstrated a strong ability to identify immune quiescence in stable patients post kidney transplantation, with a negative predictive value of 94% when both tests are negative and a positive predictive value of 89% for subclinical rejection when both tests are positive [11,12].

Because of the strong “rule out” capabilities of OmniGraf, it would be an ideal complement to real-time ADE knowledge and eGFR awareness to help guide clinicians’ decisions to adjust (or not) the immunosuppressive regimen and help provide a dialogue between patients and clinicians on the risk-benefit of medication adjustments. With its ability to both “rule out” and “rule in” subclinical rejection, it would also be an ideal tool to help monitor patients during and after medication adjustments. Allowing patients to express their ADEs and the impact they have on their lives, along with frank discussions with clinicians and risk-benefit of medication adjustments, may help increase patient engagement and activation. This is a key goal to help minimize the burden of symptoms and increase life participation [13].

Therefore, the aim of this study is to evaluate change in renal function over time in kidney transplant recipients who are undergoing OmniGraf monitoring in conjunction with patients’ medication-related symptom burden (MRSB) monitoring.

## Methods

### Study Design

CLARITY is a 3-year prospective, multisite, observational study of 2000 participants with a matched control, measuring the impact of real-time patients’ MRSB and the OmniGraf biomarker panel on change in renal function over time. Approximately 50 sites will be targeted to enroll participants. The targeted sites will include transplant centers and large community nephrology practices with large populations of kidney transplant recipients who fall within the time line of 3 months to 2 years post transplantation. Sites will be limited to 200 participants to limit center effects in our outcomes.

### Ethical Considerations

The study is under review by the Central Institutional Review Board (Pro00067364) as well as local institutional review boards (IRBs) at some sites (depending on local IRB requirements) and conforms to the ClinicalTrials.gov guidelines.

### Aims

The primary objective is to evaluate change in renal function over time in kidney transplant recipients who are undergoing OmniGraf monitoring in conjunction with MRSB monitoring. A secondary objective of this study is to identify the impact of OmniGraf use, in conjunction with patient-reported MRSB, as part of clinical care on patient quality of life and patient and graft survival.

### Recruitment, Screening, and Enrollment Procedures

Adult (≥18 years old) kidney transplant recipients between 3 months and 2 years post transplantation, who meet the study eligibility criteria will be identified in accordance with local site IRB-approved practices and approached by research personnel for consideration for participation. Potential participants will be required to go through an informed consent process and complete an informed consent document to ensure they understand the goals, risks, and potential benefits of the study before any research-related activities are carried out.

### Eligibility

#### Inclusion Criteria

Participants must be adult (≥18 years of age) recipients of a primary or subsequent kidney transplant, between 3 months and 2 years post transplantation, selected by their provider to undergo OmniGraf testing as part of posttransplantation care, and provide written informed consent and Health Insurance Portability and Accountability Act authorization.

#### Exclusion Criteria

Patients who are recipients of a combined organ transplant with an extrarenal or islet cell transplant, previous recipients of a nonrenal solid organ or islet cell transplant, those known to be pregnant, those infected with HIV, those who have active BK nephropathy, those who have nephrotic range proteinuria, or those who are participating in other biomarker clinical trials at the time of assessment will be excluded from participation.

### Statistical Analysis

#### Sample Size Requirements

Sample size was determined using an SAS macro program, %GFR_Slope_Power, developed by Vonesh et al [14] specifically for the purpose of determining sample size or power estimates for comparing slopes between 2 treatment groups based on the linear spline mixed-effects model. An Assumption of eGFR slope change over 3 years in standard care was adopted in accordance with Vincenti et al [15]. Collectively, it is estimated that a sample of 450 patients per protocol would be required to detect a minimum total slope difference at 1.08 mL/minute/1.73 m^2^/year with 84% power, assuring that the study will be powered for a clinically meaningful difference of 5 mL/minute/1.73 m^2^/year. Conservatively assuming a 50% dropout rate and 50% loss to follow-up or missing data for end of follow-up, for this analysis, a sample size of 2000 participants should be sufficient to demonstrate a clinically significant difference in the primary outcome. Annual and as-needed review by an advisory board will halt enrollment when it is predicted that the sample size will be met.

#### Primary Analysis

The primary outcome variable defined is a change in the slope of eGFR in participants enrolled in the study compared to that of a matched cohort. Matching variables will include sex, race, time from transplant, living or deceased donor, and baseline eGFR.

eGFR will be summarized using descriptive statistics by study visit. Plots will be used as a general guideline to assess the functional relationship of eGFR over time for modeling purposes. Change in eGFR across time will be modeled using a linear mixed-effects (random intercept random slope) model.

#### Secondary Analysis

Secondary outcomes include continuous outcomes, categorical outcomes, and time-to-event outcomes. Categorical outcomes will be evaluated using a chi-square test or the Fisher exact test, when appropriate. Death-censored graft loss will be evaluated using a Cox cause-specific hazards model. A cumulative incidence curve will be used as with 95% CIs using the cumulative incidence function. Graft and patient survival will be estimated using the Kaplan-Meier method. Factors associated with the risk of graft loss and patient death will be evaluated using a Cox extended hazards model. The effect of TruGraf test results and TRAC test results on the risk of graft loss and patient death will be incorporated in the model as time-varying covariates. Patient-reported outcome measures will be assessed using scoring tools from the Patient-Reported Outcomes Measurement Information System (PROMIS) group. Cut point methods will be used descriptively for each time point based on thresholds known by the PROMIS group at that time or linkages to legacy measures, when appropriate. Comparison across time points will be carried out using Meaningful Change Methods for each PROMIS measure based on information and guidance from the PROMIS group.

### Resources and Biomarker Testing

Participants will be provided with access to a method of reporting real-time MRSB via smartphone app or the internet and instructed to answer the MRSB questionnaire whenever they are experiencing ADE or prior to clinic visits (Textbox 1). The MRSB questionnaire is based on side effects that have been considered in previously validated side effect measures and is based on a questionnaire used in a mobile health analysis that was found to reduce hospitalizations and grade 3 or higher ADEs [9].

Physicians will have access to a portal that will include an easy-to-read report of responses, simultaneously prioritizing frequent MRSB that the participant considers at least moderately troubling. Participants will also undergo OmniGraf biomarker testing based on the frequency of their standard of care laboratory testing (Table 1). Laboratory testing data are not mandated and will be collected and entered into the electronic case report forms if available in the patient record.

**Medication-related symptom burden questionnaire.**

**1. Do you have trembling hands?**

- Not at all
- Very little
- Sometimes
- Often
- All the time

**1A. How troublesome is it?**

- Not at all
- Very little
- Moderately troubling
- Very troubling
- Extremely troubling

**2. Do you have trouble falling or staying asleep?**

- Not at all
- Very little
- Sometimes
- Often
- All the time

**2A. How troublesome is it?**

- Not at all
- Very little
- Moderately troubling
- Very troubling
- Extremely troubling

**3. Are you having trouble with unplanned changes in weight?**

- Not at all
- Very little
- Sometimes
- Often
- All the time

**3A. How troublesome is it?**

- Not at all
- Very little
- Moderately troubling
- Very troubling
- Extremely troubling

**4. Do you have loss of interest in or the ability to perform sex?**

- Not at all
- Very little
- Sometimes
- Often
- All the time

**4A. How troublesome is it?**

- Not at all
- Very little
- Moderately troubling
- Very troubling
- Extremely troubling

**5. Do you have nausea?**

- Not at all
- Very little
- Sometimes
- Often
- All the time

**5A. How troublesome is it?**

- Not at all
- Very little
- Moderately troubling
- Very troubling
- Extremely troubling

**6. Do you have diarrhea?**

- Not at all
- Very little
- Sometimes
- Often
- All the time

**6A. How troublesome is it?**

- Not at all
- Very little
- Moderately troubling
- Very troubling
- Extremely troubling

**7. Do you have mood changes or feelings of depression?**

- Not at all
- Very little
- Sometimes
- Often
- All the time

**7A. How troublesome is it?**

- Not at all
- Very little
- Moderately troubling
- Very troubling
- Extremely troubling

**8. Do you have nervousness or anxiety?**

- Not at all
- Very little
- Sometimes
- Often
- All the time

**8A. How troublesome is it?**

- Not at all
- Very little
- Moderately troubling
- Very troubling
- Extremely troubling

**9. Do you have difficulty concentrating or remembering to do things?**

- Not at all
- Very little
- Sometimes
- Often
- All the time

**9A. How troublesome is it?**

- Not at all
- Very little
- Moderately troubling
- Very troubling
- Extremely troubling

**10. Do you have feelings of anger or irritability?**

- Not at all
- Very little
- Sometimes
- Often
- All the time

**10A. How troublesome is it?**

- Not at all
- Very little
- Moderately troubling
- Very troubling
- Extremely troubling

**11. Do you have headaches?**

- Not at all
- Very little
- Sometimes
- Often
- All the time

**11A. How troublesome is it?**

- Not at all
- Very little
- Moderately troubling
- Very troubling
- Extremely troubling

**Table 1:** Schedule of assessments.

Enrollment or months post enrollment			Baseline visit (days –90 to 0)		3		6		9		12		15		18		21		24		27		30		33		36		Unscheduled visit		At workup for referral to transplant center
Visits	Details																														
Informed consent	Prior to any study-related procedures	✓																													
Assessment inclusion or exclusion criteria			✓																												
Participant’s demographics	Date of birth, sex, race, height, weight, and date of transplant	✓																													
Transplant information	Donor type and cause for renal failure	✓																													
Chemistry panel	Serum creatinine	✓		SOC^a^		SOC		SOC		SOC		SOC		SOC		SOC		SOC		SOC		SOC		SOC		SOC		SOC		SOC	
Immunosuppression medications	Name and changes in dose or medication			✓		✓		✓		✓		✓		✓		✓		✓		✓		✓		✓		✓		✓		✓	
Assessment of clinical events	Rejections, infections, graft Loss, and death	✓		✓		✓		✓		✓		✓		✓		✓		✓		✓		✓		✓		✓		✓		✓	
OmniGraf results	TruGraf and TRAC test results (if SOC laboratory tests are performed)	✓ (day 0)		✓		✓		✓		✓		✓		✓		✓		✓		✓		✓		✓		✓		✓		✓	
Patient’s medication-related symptom burden			✓		✓		✓		✓		✓		✓		✓		✓		✓		✓		✓		✓		✓		✓		✓
Biopsy information	Surveillance and for-cause: in case of rejection, type and grade			SOC		SOC		SOC		SOC		SOC		SOC		SOC		SOC		SOC		SOC		SOC		SOC		SOC		SOC	
Patient-reported outcomes	PROMIS^b^-29, PROMIS Self-Efficacy, and PROMIS Depression Scale	✓								✓								✓								✓					
Provider satisfaction			✓								✓								✓								✓				

^a^SOC: standard of care.

^b^PROMIS: Patient-Reported Outcomes Measurement Information System.

**Figure 1:**
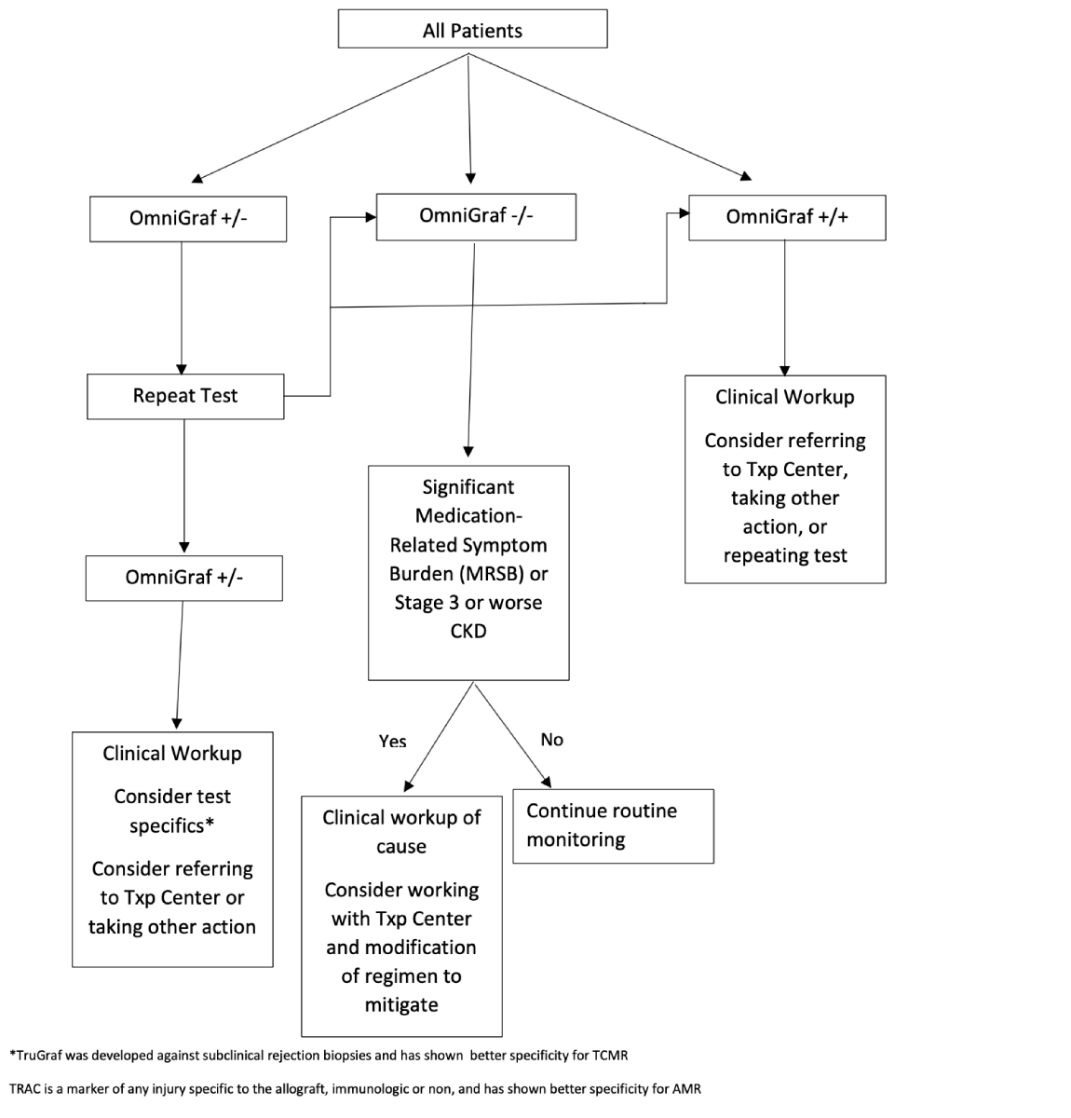
Suggested framework for integrating OmniGraf and patients' medication-related symptom burden monitoring into clinical care. AMR: antibody mediated rejection; CKD: chronic kidney disease; TCMR: t-cell mediated rejection; Txp: transplant.

Physicians can use the MRSB and OmniGraf results as they see fit, along with other clinical laboratory tests and information; however, they will be provided with a potential framework for integrating the information into their practice (Figure 1). In short, clinicians will see participants and review the MRSB portal and clinical laboratory tests, including OmniGraf. If the clinician notes that the participant has less than ideal renal function or has an MRSB that is troubling him/her, a discussion can be had between the clinician and participant. A clinical workup for the renal function or ADE can be performed to identify other treatable causes. If it is determined that the ADE (including renal function) is caused by or exacerbated by medications that the participant is taking, the laboratory findings and OmniGraf biomarker results can be used within the clinician-participant discussion to help determine the risks and benefits of adjusting medications to mitigate the ADE. OmniGraf results can also provide additional information to clinicians regarding participants who do not have a low eGFR or ADE, assisting in their clinical decision-making or identifying participants who may be at risk of subclinical inflammation.

## Results

### Results Overview

The primary outcome measure of this study will be the slope change in renal function over time in kidney transplant recipients who are undergoing OmniGraf monitoring in conjunction with MRSB monitoring. Secondary outcome measures include the PROMIS Self-Efficacy for Managing Chronic Conditions–Managing Medications and Treatment–Short Form 4a; the PROMIS-29 Profile (version 2.1); the PROMIS Depression Scale, hospitalizations—subcategorized for hospitalizations owing to infections; treated rejections, MRSB, and the proportion of participants with overall graft survival at year 3 post transplantation; graft loss or death during the 3-year study follow-up period; and change in provider satisfaction.

### Study Endpoint Definitions and Assessment Plan

The following will be used to define and assess events within this study.

The primary endpoint is a comparison of the slope change in eGFR from baseline to the end of follow-up between the study participants and a matched control group. eGFR will be calculated using the 4-variable Modification of Diet in Renal Disease equation [16], with a sensitivity analysis performed using the Chronic Kidney Disease Epidemiology Collaboration equation [17]. Routine serum creatinine concentrations, which are measured as a part of usual care, will be utilized to estimate the GFR at baseline and at months 12, 24, and 36 post enrollment for assessments. The slope in eGFR change will be calculated for the entire follow-up for the primary outcome, with subanalyses performed between the other described time points. The matched control group will be a propensity-matched cohort from the US Medicare database.

Furthermore, we will compare the PROMIS Self-Efficacy for Managing Chronic Conditions–Managing Medications and Treatment–Short Form 4a scores at the end of follow-up relative to baseline [18]. This will measure changes in self-efficacy within the participant population—the belief that one can carry out a behavior necessary to reach a desired goal, even when a situation contained unpredictable and stressful elements. The study coordinator will provide access to the survey at baseline and at months 12, 24, and 36 post enrollment. Subanalyses will be performed among all time points.

We will then compare the PROMIS-29 Profile (version 2.1) scores at the end of follow-up relative to baseline [19]. This will measure changes in overall quality of life and satisfaction. The study coordinator will provide access to the survey at baseline and at months 12, 24, and 36 post enrollment. Subanalyses will be performed among all time points.

This will be followed by a comparison of the PROMIS Depression Scale scores at the end of follow-up relative to baseline [20]. This will measure changes in symptoms of depression. The study coordinator will provide access to the survey at baseline and at months 12, 24, and 36 post enrollment. Subanalyses will be performed among all time points.

Hospitalizations, subcategorized for hospitalizations resulting from infections, will be compared between study participants and a matched control group. Study coordinators will obtain information on hospitalizations and causes at all follow-up events. This will be compared to hospitalizations and causes documented within the US Medicare database for a propensity-matched control group. Hospitalizations will be defined as admission to hospital with at least one overnight stay. Length of hospital stay will also be recorded.

MRSB, as defined as the change in the number and severity of ADE self-reported by study participants, will be recorded from the end of follow-up relative to baseline.

Overall graft failure, defined as return to chronic dialysis, transplant nephrectomy, retransplantation, or death, will also be recorded. The study coordinator capturing clinical event data will review the medical record at intervals in accordance with the schedule of evaluations to determine if a study participant has developed graft failure. The timing and cause of each graft loss will be recorded for comparative analysis with the propensity-matched external cohort. Patient death will be captured in a similar manner, with timing and cause recorded as well.

The anticipated first enrollment of participants in the study is October 2022, with data analysis and publication expected in October 2027.

## Discussion

Owing to the comorbidities and toxicities associated with posttransplantation care, including immunosuppressive medication regimens, the care of kidney transplant recipients is highly complex and fraught with clinical conundrums. Founded on data demonstrating high rule-out capabilities, we hypothesize that the use of OmniGraf in conjunction with patients’ MRSB monitoring will provide a promising and innovative approach to improving posttransplantation renal function. The ultimate goal of the research is to demonstrate how patients, clinicians, and biomarkers can work harmoniously to optimize and personalize posttransplantation care.

## Abbreviations

ADE: adverse drug event
CKD: chronic kidney disease
CLARITY: CLinical Utility of the omnigrAf biomarkeR Panel In The Care of kidneY Transplant Recipients
eGFR: estimated glomerular filtration rate
IRB: institutional review board
MRSB: medication-related symptom burden
PROMIS: Patient-Reported Outcomes Measurement Information System
